# Anti-Inflammatory Response of New Postbiotics in TNF-α/IFN-γ-Induced Atopic Dermatitis-like HaCaT Keratinocytes

**DOI:** 10.3390/cimb46060364

**Published:** 2024-06-17

**Authors:** Yoo-Kyung Kim, Minji Cho, Dae-Jung Kang

**Affiliations:** MNH Bio Co., Ltd., Dongtan-Biz-Tower 609, Dongtancheomdansaneop 1-ro, Hwaseong-si 18469, Gyeonggi-do, Republic of Korea; ykkim@mnhbio.com (Y.-K.K.); mjcho@mnhbio.com (M.C.)

**Keywords:** atopic dermatitis, anti-inflammatory, cytokines, chemokines, HaCaT keratinocytes, TNF-κB, postbiotics

## Abstract

This study examines the synergistic interaction between the immunomodulatory functions of lactic acid bacteria postbiotics and the anti-inflammatory properties of *Smilax china* L. extract through a combined fermentation process. Using atopic dermatitis (AD) as a model, characterized by an immune imbalance that leads to skin inflammation, we developed a fermented product, MB-2006, and compared its effects to those of the heat-killed probiotics *Lactobacillus acidophilus* (LAC) and *Lactobacillus rhamnosus* (LRH). Our experiments focused on elucidating the mechanism of action of MB-2006 in AD-like HaCaT keratinocyte cells, particularly its impact on the NF-κB pathway, a pivotal regulator of inflammation. MB-2006 proved more effective in reducing inflammation markers, such as IL-4 and thymic stromal lymphopoietin (TSLP), and in inhibiting NF-κB activation compared to LAC and LRH. Significantly, MB-2006 also reduced the expression of thymus- and activation-regulated chemokine (TARC), highlighting a synergistic effect that enhances its therapeutic potential. These results suggest that the combined fermentation of *Smilax china* L. extract with lactic acid bacteria enhanced both the anti-inflammatory and immunomodulatory effects, presenting a promising integrative approach to treating conditions like AD. Further studies are needed to validate these results in clinical settings and fully explore the potential of this synergistic fermentation process.

## 1. Introduction

Atopic dermatitis (AD) is a prevalent and debilitating inflammatory skin disease that significantly diminishes quality of life [1]. AD, known as a recurrent chronic inflammatory pruritic disease, is associated with a variety of immune and genetic factors, making tailored treatment difficult [2,3]. It involves complex immunologic reactions and genetic predispositions, with an increasing burden recognized in pediatric and adult populations worldwide over the past decade [4,5]. Although both prevention and treatment of AD are urgent, the use of immune modulators and biological agents for AD treatment cause significant discomfort to patients due to frequent side effects, such as conjunctivitis and headache [6]. As the demand for safe, side-effect-free preventive agents increases, the use of products related to biological agents such as lactic acid bacteria or natural products with immunomodulatory functions is gaining great attention.

Natural products containing significant amounts of polyphenolic compounds, such as flavonoids, alkaloids, terpenes, and glycosides, are known to have potential pharmacological effects as regulators of multiple signaling pathways rather than a single mode of action. Based on this, there is some scientific proof of safety and effectiveness in atopic dermatitis [7], particularly with various natural products that have been shown to reverse pathological changes in AD-like dermatitis in animal and cell experiments [7,8]. Among these, *Smilax china* L., a member of the *Smilacaceae* family, is widely distributed worldwide in tropical and temperate regions, especially in East Asia [9]. Recently, many studies have shown the anti-inflammatory effects of *Smilax china* L. leaves, which contain a significant amount of polyphenols [10] and is one such natural product that has shown promising anti-inflammatory properties. These bioactive compounds are known to inhibit various inflammatory mediators and pathways, thereby reducing inflammation and improving overall skin health, *Smilax china* L. has been traditionally used in East Asian medicine for its purported health benefits, including its anti-inflammatory and detoxifying properties.

The outermost layer of the skin, primarily composed of keratinocytes, acts as a barrier against environmental insults and plays a crucial role in the immunological integrity of the skin. Dysfunction of this barrier is central to the pathogenesis of AD, characterized by a dysregulated immune response, leading to excessive inflammation and pruritus [11]. These AD symptoms can worsen the inflammatory response in the skin by over-secreting chemokines (macrophage-derived chemokine (MDC), thymus- and activation-regulated chemokine (TARC)) and inflammatory cytokines (thymic stromal lymphopoietin (TSLP), IL-4, IL-25, and IL-33) in keratinocytes [12]. Excessive secretion of these cytokines and chemokines implies a malfunction in the regulatory mechanisms of TNFα and IFN-γ, which determine their secretion. By modulating these regulatory mechanisms, it is anticipated that the secretion of both cytokines and chemokines can be reduced, thereby alleviating the symptoms of AD.

Probiotics are live microorganisms that, when consumed in sufficient quantities, are widely recognized for their health-promoting effects and are generally regarded as safe [13]. These beneficial microorganisms have been extensively documented for their ability to not only restore the balance of the gut microbiome but also significantly improve skin health [14]. This is particularly relevant in the treatment and management of various skin disorders, including atopic dermatitis (AD), ichthyosis, acne, and psoriasis [15]. The enhancement of skin health is primarily attributed to the metabolites, cell wall components, and non-viable cells of probiotics, with short-chain fatty acids (SCFAs) from the supernatant of these organisms identified as key beneficial agents [15].

Amid these concerns, the scientific community has turned its attention to postbiotics. Defined by the International Scientific Association of Probiotics and Prebiotics (ISAPP) as preparations of inanimate microorganisms and/or their components that confer health benefits. Postbiotics include heat-killed bacteria, cell-free supernatants, and purified microbial components that maintain the beneficial properties of probiotics while ensuring safety and stability [16]. These elements are processed through methods such as heat treatment, chemical inactivation using agents like formalin, irradiation with gamma or ultraviolet light, and mechanical disruption through sonication [17]. Such methods ensure the non-viability of probiotics while maintaining their beneficial properties for safe pharmaceutical applications [18]. These postbiotic preparations have demonstrated various health benefit, including anti-inflammatory, immunomodulatory, and antioxidant effects, making them a promising alternative for the treatment of AD and other inflammatory conditions.

Looking at recent research trends, research into the regulation of microbial communities is increasingly shifting towards postbiotics. Numerous studies have demonstrated the potential of natural products and probiotics to bolster immune regulation in AD-like keratinocyte models, although the clinical outcomes have often not met expectations [15,19,20]. Postbiotics are emerging as a promising alternative to address these shortcomings. However, research into the establishment of AD in keratinocytes and the effectiveness of postbiotics in these models remains limited. Further investigation is required to elucidate the role of postbiotics in improving AD treatment efficacy.

Recent research has illuminated the complex mechanisms of immune dysregulation associated with AD [21]. As already mentioned, postbiotics are attracting attention due to their immunomodulatory and anti-inflammatory properties, which promise not only to treat AD but also to enhance gut health and overall well-being. Additionally, studies have indicated that active compounds from plants, particularly those rich in diverse flavonoids, may mitigate AD symptoms. Consequently, employing a fermentation process that uses flavonoid-rich plants in conjunction with lactic acid bacteria known for their strong anti-inflammatory and immune-modulating effects and incorporating non-viable microbial cells, may offer a robust approach to tackle the multifaceted challenges of AD. Furthermore, fermentation metabolites such as lactic acid and acetic acid are crucial for enhancing these effects [18]. The fermentation process with lactic acid bacteria significantly enhances the anti-inflammatory properties of *Smilax china* L. extract by altering its biochemical composition and producing key metabolites such as bioactive peptides, phenolic acids, and free flavonoids.

Here, we established a well-known AD in vitro model and we investigated the AD improvement effect of MB2006, prepared through *Smilax china* L. fermentation by a *Lactobacillus* strain, for its inflammation relief and immune modulating functions. We aimed to verify the efficacy of our postbiotic products. This model allowed us to investigate the specific mechanisms by which MB-2006 exerts its effects, particularly its impact on the NF-κB pathway, a key regulator of inflammation.

## 2. Materials and Methods

### 2.1. Preparation of Postbiotics (MB-2006)

*Smilax china* L. leaves (SCL) were collected at Uiryong-gun, southern region of Korea, and were dried. The dried samples were pulverized into fine powder using a stainless steel blender (RT-08; MHK Co., Seoul, Republic of Korea). The strain used in this study was *Lactobacillus acidophilus* (KCTC15475BP, MNH Bio Co., Ltd., Gyeonggi-do, Republic of Korea), and it was cultured in MRS broth (BD Biosciences, San Jose, CA, USA) with 2% (*w*/*v*) fine powder of *Smilax china* L. at 37 °C for 48 h. After 48 h, the fermented cultures were heat-killed at 121 °C for 15 min and centrifuged at 1200× *g* at 4 °C for 15 min, and the bacterial pellets were diluted in the supernatant of the fermented culture to a final density (MB-2006). *Lactobacillus acidophilus* (KCTC 15475BP, LAC) and *Lactobacillus rhamnosus* UBC90 (MNH Bio Co., Ltd., LRH) were isolated from Gimchi. They were used as a positive control and were cultivated in MRS broth (BD Biosciences, USA) at 37 °C for 48 h. After 48 h, the fermented cultures were heat-killed at 121 °C for 15 min and centrifuged at 1200× *g* at 4 °C for 15 min, and the bacterial pellets were diluted in PBS to a final density.

### 2.2. Cell Line and Cell Culture

HaCaT keratinocytes (Korea Cell Line Bank, Seoul, Republic of Korea) were cultured in DMEM (Gibco, Grand Island, NY, USA) containing 10% FBS (Gibco) and 1% penicillin and streptomycin (Gibco) [4]. The cells were cultured at 37 °C under 5% CO_2_ conditions.

### 2.3. Cell Viability

First, HaCaT keratinocytes were seeded at a density of 1 × 10^5^ cells/well in 96-well plates and cultured for 24 h. To evaluate cell toxicity, heat-killed MB-2006 was treated at 1 × 10^7^, 1 × 10^8^ and 1 × 10^9^ cells/mL of the cultured supernatant for 24 h. For comparison, heat-killed LAC and LRH were also treated at the same cell concentrations as heat-killed MB-2006. All postbiotic-treated samples were incubated in a CO_2_-incubator at 37 °C for 24 h. After incubating cells, each cell viability was determined using the MTT(3-(4,5-dimethylthiazol-2-yl)-2,5-diphenyltetrazolium bromide) assay, a colorimetric assay for reflecting the number of viable cells present [5]. For detecting colorimetric change, absorbance at 540 nm was measured using a microplate spectrophotometer (Bioteck, Daejeon, Republic of Korea).

### 2.4. Measurement of Cytokines and Chemokines in TNF-α/IFN-γ-Induced HaCaT Keratinocytes

The HaCaT cells were seeded in 96-well plates at a density of 1 × 10^5^ cells/well and incubated at 37 °C for 24 h. Then, the cultured HaCaT cells were treated with MB-2006 and other postbiotics (LAC and LRH) at concentrations of 1 × 10^7^, 1 × 10^8^ and 1 × 10^9^ cells/mL for 1 h. The pretreated HaCaT cells were stimulated with TNF-α (10 ng/mL; R&D Systems, Neapolis, MN, USA) and IFN-γ (10 ng/mL; R&D Systems, Minneapolis, MN, USA) to induce an AD-like physiological state.

Cytokine levels in each supernatant were measured by the enzyme-linked immunosorbent assay (ELISA) method. The levels of cytokines (IL-4, IL-13, IL-10, and IL-31) were measured using a commercial cytokine ELISA kit (BD Biosciences, San Jose, CA, USA) for each cytokine. The levels of chemokines (TSLP, TARC and MDC) were also measured using a commercial chemokine ELISA kit (BD Biosciences, San Jose, CA, USA) for each chemokine.

### 2.5. Western Blotting

For protein extraction from HaCaT cells, protein lysates were prepared using a radioimmunnoprecipitation assay (RIPA) cell lysis buffer (HanLab, Seoul, Republic of Korea), including phosphatase and protease inhibitors (Roche, Basel, Switzerland). To evaluate NF-κB p65 and phosphorylated NF-κB p65, they were resolved on 10% polyacrylamide gels, transferred to nitrocellulose membranes (Amersham Pharmacia Biotech, Piscataway, NJ, USA). For detection of the target protein, the appropriate primary (anti-p65 (Cat. No. 8242S), anti-phospho-p65 (Cat. No. 3033S), anti-β-actin (Cat. No. 3700S), CST) and secondary (anti-mouse IgG, HRP-linked antibody (Cat. No. 7076P2), CST) antibodies were used. Membranes were visualized using an enhanced ChemiDoc MP imaging system (BioRad, Hercules, CA, USA), and quantified using ImageJ software Version 6.1.

### 2.6. Statistical Analysis

The experiments were carried out in triplicates for each combination. Experimental data were analyzed by the statistical package GraphPad Prism 6 (GraphPad Software, Inc., La Jolla, CA, USA). Data are presented as means ± standard deviation (SD). Statistical analysis was performed by applying two-way ANOVA, followed by Tukey’s post-hoc test for multiple comparisons. A *p*-value of *p* ≤ 0.05 was considered significant. (* *p* ≤ 0.05; ** *p* ≤ 0.01; *** *p* ≤ 0.001).

## 3. Results

### 3.1. Cytotoxicity of MB-2006 and Other Strains

**Figure 1:**
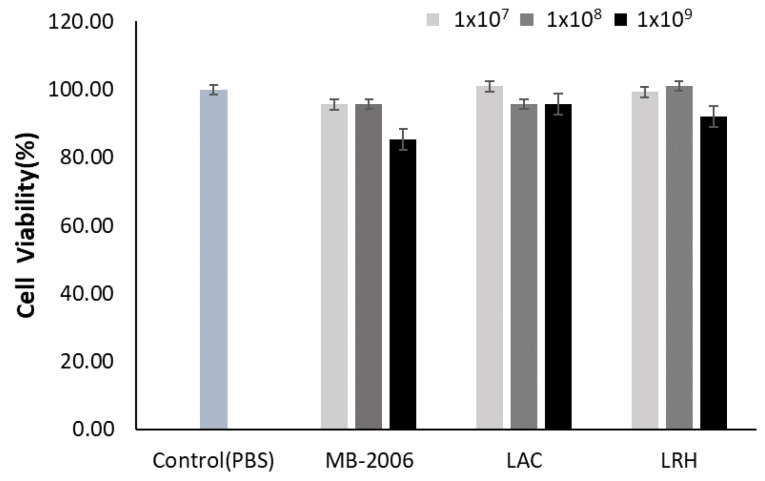
Effect of MB-2006 and other strains on cell viability. The HaCaT cells were treated with MB-2006 (1 × 10^7^, 1 × 10^8^ and 1 × 10^9^ cells/mL of culture supernatant), and LAC and LRH (1 × 10^7^, 1 × 10^8^ and 1 × 10^9^ cells/mL of PBS). The data are presented as the mean ± SEM (n = 3).

HaCaT keratinocytes were incubated with various concentrations of MB-2006, LAC, and LRH to investigate their toxic effects. Concentrations of 1 × 10^9^ cell/mL of MB-2006 and LRH appeared to be slightly cytotoxic to HaCaT keratinocytes, but all tested concentrations of postbiotics did not exhibit serious cytotoxicity compared with the PBS control (Figure 1). Based on these results, subsequent experiments related to efficacy were performed at the same concentrations (1 × 10^7^~1 × 10^9^ cells/mL). The occurrence of mild cytotoxicity in the high-concentration treatment group (1 × 10^9^ cells/mL) is presumed to be an interference effect due to the high concentration of residual cell components, characteristic of postbiotics.

### 3.2. Inhibition of Cytokines Related to Immune Response by MB-2006 in TNF-α/IFN-γ-Induced HaCaT Keratinocytes

Pro-inflammatory cytokines, pivotal in the context of AD, are known for their role in promoting allergic reactions and triggering various biological effects within the immune system [6]. These cytokines exacerbate the inflammatory response and contribute to the immune dysregulation typically observed in AD cases. The scientific investigation into natural products and lactic acid bacteria has primarily focused on their roles in immune regulation. A recent study utilized a TNF-α/IFN-γ–induced AD model to simulate a more intricate immune response, which closely resembles the cytokine and chemokine secretion profiles found in AD patients’ keratinocytes, providing a realistic reflection of immune-modulatory dynamics in AD [11].

AD, a complex inflammatory skin condition characterized by defects in the epidermal barrier and a dysregulated Th2 immune response, is not fully understood [12]. Research indicates that human keratinocytes respond more robustly to Th1-derived lymphocytes than to Th2-derived ones in terms of chemokine release [22], highlighting an elaborate inflammatory network essential in driving AD-like skin alterations. Furthermore, IL-4 is known to influence Th1-type responses, including those involved in antigen-induced autoimmune diseases [23]. These findings suggest that Th1 and Th2 cytokines participate in a complex inflammatory network driving AD-like alterations. Consequently, the TNF-α/IFN-γ-induced HaCaT cell line, which presents a mixed chronic and aggravated status of AD, was used to evaluate the efficacy of functional foods or drugs [12,24].

**Figure 2:**
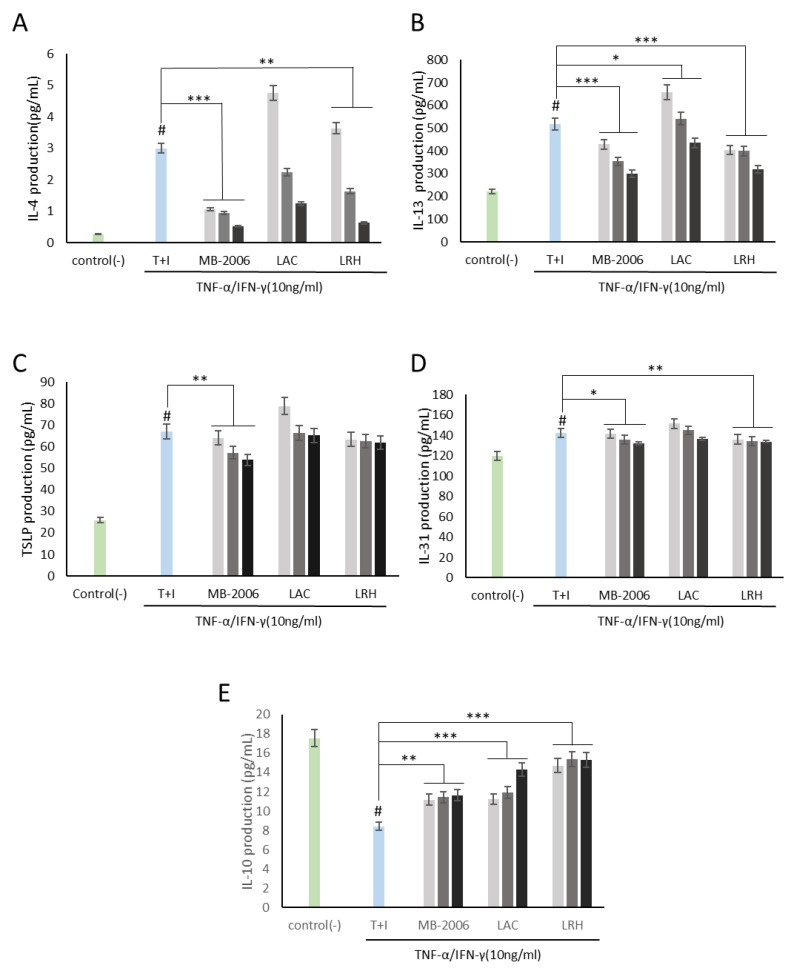
MB-2006 and other strains affect the expression of cytokines in TNF-α/IFN-γ-induced HaCaT keratinocytes. Evolution of cytokines in concentration at 1 × 10^7^ bacteria/mL (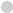), 1 × 10^8^ bacteria/mL (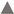) and 1 × 10^9^ bacteria/mL (■). (**A**) Interleukin (IL)-4, (**B**) IL-13, (**C**) thymic stromal lymphopoietin(TSLP), (**D**) IL-31, and (**E**) IL-10. The bars indicate the mean ± SD, and significant differences are shown in comparison to T + I (TNF-α/IFN-γ only treated group). # *p* < 0.001 vs. the negative control; * *p* < 0.05, ** *p* < 0.01, *** *p* < 0.001 vs. T + I.

Using this AD-like HaCaT cell line, we investigated how MB-2006 postbiotics may control immune biomarkers associated with AD responses. As shown in Figure 2A,B, both IL-4 and IL-13 levels in HaCaT keratinocytes were significantly elevated by TNF-α/IFN-γ induction, establishing a reasonable AD model. Based on this established model, immunomodulatory functions were investigated. IL-4 and IL-13, along with IL-3, IL-5, and IL-9, belong to the Th2 family of cytokines [25]. These cytokines are key mediators of allergic inflammation. IL-4 and IL-13 have important immunomodulatory activities and affect various immune cells, such as keratinocytes. Recent findings suggest that IL-4 and IL-13 play an important role in the downregulation of inflammatory processes underlying atopic dermatitis pathology and may favorably regulate the disease course [21]. As shown in Figure 2A,B, the increased IL-4 and IL-13 were significantly alleviated by treatment with MB-2006 and other postbiotics. Among the tested postbiotics, MB-2006 was found to be more effective in stabilizing cytokines compared to LAC and LRH (*p* < 0.001).

Additionally, both TSLP, related to the initiation of the inflammatory response [12], and IL-31, related to the induction of itching [26], were elevated in our AD model induced by TNF-α/IFN-γ stimulation, but were partially alleviated (Figure 2C,D). According to Nygaard et al., clinical studies have shown significantly increased expression levels of TSLP and IL-31 in the serum of AD patients compared to controls [27]. Within this framework, these mitigated TSLP and IL-31 may be key biological indicators for improving AD symptoms.

IL-10 is a cytokine with potent anti-inflammatory properties that plays a central role in limiting host immune responses to pathogens, thereby preventing damage to the host and maintaining normal tissue homeostasis [28]. As shown in Figure 2E, IL-10, which has anti-inflammatory properties and can help regulate the immune response, was restored in proportion to concentration by all postbiotics, demonstrating their potential in improving IL-10 dysregulation associated with the risk of developing many autoimmune diseases.

### 3.3. Inhibition of Chemokines Related to Immune Response by MB-2006 in TNF-α/IFN-γ-Induced HaCaT Keratinocytes

**Figure 3:**
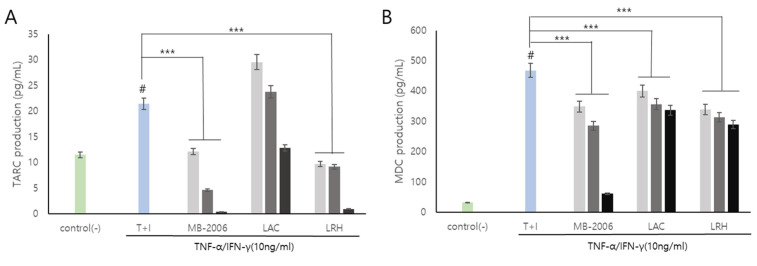
MB-2006 and other strains affect the expression of chemokines in TNF-α/IFN-γ-induced HaCaT keratinocytes. Evolution of chemokines (TARC, and MDC) in concentration at 1 × 10^7^ bacteria/mL (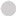), 1 × 10^8^ bacteria/mL (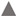) and 1 × 10^9^ bacteria/mL (■). (**A**) Thymus and activation-regulated chemokine(TARC), and (**B**) Macrophage-derived chemokine(MDC). The bars indicate the mean ± SD, and significant differences are shown in comparison to T + I (TNF-α/IFN-γ only treated group). # *p* < 0.001 vs. the negative control; *** *p* < 0.001 vs. T + I.

*Smilax china* L. contains several bioactive components such as flavonoids, polyphenols, steroid saponins, and polysaccharides, and has shown potential against various pathologies due to its anti-inflammatory properties. In particular, chemokines such as TARC and MDC are inflammatory markers that also show correlations with AD symptoms and are known to be important mediators of inflammation and immune responses [29]. Additionally, it has been reported that the regulatory function of various inflammatory chemokines is amplified through lactic acid bacteria fermentation. The induction of TNF-α/IFN-γ leads to the upregulation of numerous cytokines (such as members of the IL family and TSLP) and chemokines (including TARC and MDC) in keratinocytes [30]. TARC and MDC, serving as specific ligands for the CC motif chemokine ligand 4 (CCR4) expressed by Th2 cells, have been associated with the pathogenesis of AD [15]. In a TNF-α/IFN-γ induced AD-like model using keratinocytes, our results showed that the levels of the inflammatory chemokines TARC and MDC were significantly elevated (Figure 3). These chemokines are pivotal in exacerbating the immune response and contributing to the chronic and persistent nature of AD, thereby making them essential therapeutic targets for effective treatment strategies.

As is well known, AD is recognized as a complex inflammatory skin condition primarily driven by Th2 immune responses [31]. It is characterized by elevated levels of cytokines, such as IL-4, IL-5, and IL-13, hallmark features of Th2 dominance. However, the disease pathology also involves Th1 cells and innate inflammatory cytokines, such as IFN-γ and IL-12, present in chronic AD lesions. Notably, IFN-γ expression correlates with the clinical progression of AD and decreases as the condition improves, which is associated with a broader immune response. This study utilized the TNF-α/IFN-γ-induced HaCaT cell line as a model to simulate AD-like features, including changes in epidermal differentiation proteins influenced by both Th1 and Th2 cytokines (Figure 2 and Figure 3). This model effectively mimicked the inflammatory changes observed in AD, making it a pertinent choice for investigating the disease’s pathogenic mechanisms. Despite primary human epidermal keratinocytes being the ideal model for studying AD skin characteristics, immortalized keratinocytes like HaCaT cells can provide a viable alternative for in vitro assays due to their adaptability and consistent response to cytokine stimulation [12].

Upon treatment with MB-2006, the levels of TARC and MDC decreased in a concentration-dependent manner (*p* < 0.001) (Figure 3). This effect was most pronounced at a concentration of 1 × 10^9^ cells/mL, as shown in Figure 3A,B. These decreases of the levels of TARC and MDC were significantly more effective when compared to other postbiotics tested in the study, indicating the distinctive efficacy of MB-2006 in modulating inflammatory responses within this model. The potent anti-inflammatory effects of MB-2006 are likely due to the action of fermented flavonoids derived from *Smilax china* L., aligning with the well-documented anti-inflammatory capabilities of natural plant flavonoids. These results clearly indicate the synergistic effect of MB-2006 on the AD-like model and its therapeutic potential. Our findings validated the effects of postbiotic treatments in the AD model where Th1 and Th2 cytokines interact, highlighting the potential of MB-2006 to influence both innate and adaptive immune responses in the development and progression of AD.

Taken together, these results indicate that MB-2006 not only significantly alleviates AD symptoms by effectively reducing key inflammatory chemokines but also operates through a clearly defined biochemical pathway. The distinctive properties of MB-2006, especially its formulation with fermented *Smilax china* L., distinguished it from other postbiotics and showed its potential as a superior therapeutic agent in the treatment of AD. To further understand its mechanism, we investigated whether this suppression of AD symptoms was mediated through the NF-κB signaling pathway, which is known to regulate immune and inflammatory responses in Th2 [29].

### 3.4. Effect of MB-2006 on NF-κB Signaling Pathway in TNF-α/IFN-γ-Induced HaCaT Keratinocytes

AD is driven by complex inflammatory pathways where NF-κB, a key transcription factor, plays a central role. As is well known, NF-κB, a key inflammatory transcription factor, is crucial for the expression of genes related to allergies, inflammation, and immune responses by regulating cytokines [31]. This NF-κB signaling pathway is regarded as the primary pre-inflammatory pathway due to NF-κB’s role in expressing pre-inflammatory genes such as adhesion molecules, chemokines, and cytokines [32]. Cellular responses to extracellular stimuli, like bacterial or viral infections and stress, require rapid and precise signal transmission from cell-surface receptors to the nucleus [33]. These signaling pathways involve protein phosphorylation, leading to the activation of transcription factors such as NF-κB, which is essential for inflammation, immunity, cell proliferation, and apoptosis. NF-κB remains in a latent state and is activated via pathways that typically involve the phosphorylation and subsequent degradation of its inhibitor, IκB. This activation allows NF-κB to enter the nucleus and modulate gene expression, underlining its pivotal role in inflammatory responses, as observed in AD models [34].

**Figure 4:**
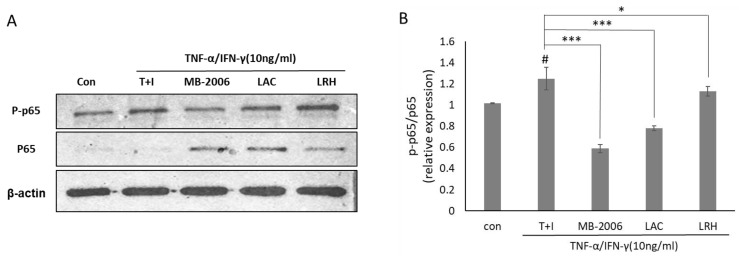
Effect of MB-2006 and other strains on TNF-α/IFN-γ-induced NF-κB activation in HaCaT cells. The protein expression of NF-κB (**A**) was normalized to non-phosphorylated protein. The bars (**B**) indicate the mean ± SD, and significant differences are shown in comparison to T + I (TNF-α/IFN-γ only treated group). # *p* < 0.001 vs. the negative control; * *p* < 0.05, *** *p* < 0.001 vs. T + I.

Since upregulation of NF-κB activity is directly associated with the inflammatory response in AD, the change in NF-κB activity in AD-like models may explain its relevance to the effects of MB-2006. To further investigate the impact of MB-2006 on the NF-κB signaling pathway in TNF-α/IFN-γ-induced HaCaT keratinocytes, it was analyzed using Western blotting (Figure 4A). These results showed increased phosphorylation of p65 following TNF-α/IFN-γ treatment, while non-phosphorylated p65 was almost undetectable. To clarify this observation, we examined the relative p-p65/p65 ratio (Figure 4B). The p-p65/p65 ratio was elevated in TNF-α/IFN-γ-induced HaCaT keratinocytes (*p* < 0.05), whereas it was decreased in samples treated with other postbiotics. Notably, the p-p65/p65 relative ratio in MB-2006-treated cells was significantly reduced (*p* < 0.001). This observation indicates a typical mechanism of action that involves downregulating the pathogenic process by reducing NF-κB activation rather than merely controlling its expression level to alleviate the inflammatory response in the AD-like model HaCaT cells. This result means that MB-2006 can mitigate NF-κB activation by dephosphorylating p65, leading to a decrease in the inflammatory response in the AD-like model HaCaT cells. Particularly, when compared to other lactic acid bacteria, the greater NF-κB reduction effect observed with MB2006 can be attributed to the synergistic effect of *Smilax china* L. fermentation. Thus, it can be concluded that MB2006 suppresses the AD response by effectively regulating chemokines and cytokines through the reduction of NF-κB activity driven by inflammation.

## 4. Discussion

The immunomodulatory properties of probiotics and postbiotics have been extensively studied, showing their potential to restore the balance of the gut microbiome and improve skin health, particularly in conditions like AD. The enhancement of skin health is primarily attributed to the metabolites, cell wall components, and non-viable cells of probiotics. SCFAs from the supernatant of these organisms are identified as key beneficial agents [35,36]. Studies have increasingly reported that probiotics can reduce the severity of AD by boosting immune regulation and strengthening the skin’s barrier functions [14,37,38]. Furthermore, a body of research has established the substantial immunomodulatory properties of probiotics, which have been harnessed in the prevention and treatment of numerous diseases.

Recent studies have highlighted the potential of natural products containing significant amounts of polyphenolic compounds, such as flavonoids, alkaloids, terpenes, and glycosides, which regulate multiple signaling pathways [7,8,9]. *Smilax china* L. is one such natural product that has shown promising anti-inflammatory properties. The bioactive compounds in *Smilax china* L., such as flavonoids and polyphenols, have demonstrated significant anti-inflammatory effects, making it a valuable candidate for developing new treatments for inflammatory conditions like AD [39].

In this study, MB-2006, a postbiotic prepared through the fermentation of *Smilax China* L. by *Lactobacillus* strains, demonstrated significant immunomodulatory and anti-inflammatory effects in TNF-α/IFN-γ-induced HaCaT keratinocytes (Figure 2 and Figure 3). The fermentation process enhances the bioavailability of flavonoids and polyphenols, which inhibit various inflammatory mediators and pathways, thereby reducing inflammation [ref]. Fermentation also produces additional metabolites such as SCFAs and bioactive peptides that further contribute to the anti-inflammatory effects. These metabolites, produced during fermentation, interact with the bioactive compounds in *Smilax china* L., enhancing their stability and efficacy.

Our results showed that MB-2006 was more effective in reducing inflammation markers, such as IL-4, TSLP, and TARC, compared to non-fermented *Smilax china* L. extract and heat-killed probiotics. This enhanced efficacy is likely due to the synergistic effects of the bioactive components in *Smilax china* L. and the metabolites produced during fermentation. The precise mechanism by which MB-2006 inhibits the NF-κB pathway in AD-like HaCaT keratinocyte cells involves multiple steps that culminate in a reduced inflammatory response characteristic of atopic dermatitis (AD) [32]. MB-2006 exerts its effects primarily through the inhibition of IκB kinase (IKK), which is responsible for phosphorylating IκBα. This inhibition prevents the degradation of IκBα, a key inhibitor that sequesters NF-κB in the cytoplasm, thereby reducing the activation of NF-κB.

Western blot analysis (Figure 4) demonstrates that MB-2006 significantly reduces the phosphorylation of p65, a subunit of NF-κB, indicating decreased NF-κB pathway activation. MB-2006 likely suppresses the activity of IκB kinase (IKK), preventing the phosphorylation and subsequent degradation of IκBα. This inhibition keeps NF-κB sequestered in the cytoplasm, limiting its ability to translocate to the nucleus and initiate the transcription of pro-inflammatory genes. The reduced p-p65/p65 ratio in MB-2006-treated cells, compared to TNF-α/IFN-γ-induced cells (Figure 4B), confirms this inhibitory effect. Additionally, MB-2006 treatment leads to a decrease in the production of pro-inflammatory cytokines, such as IL-4, IL-13, TSLP, and TARC, which are regulated by NF-κB, thereby helping to reduce inflammation and alleviate AD symptoms. Figure 2 and Figure 3 substantiate that MB-2006 significantly reduces the levels of these cytokines and chemokines in a dose-dependent manner.

In contrast, heat-killed probiotics (LAC and LRH) inhibit the NF-κB pathway through slightly different mechanisms. These probiotics often interact with pattern recognition receptors (PRRs) on the surface of immune cells, leading to downstream effects on the NF-κB pathway. Specifically, LAC and LRH modulate toll-like receptors (TLRs) and nucleotide-binding oligomerization domain (NOD) receptors, which can converge on NF-κB. The existing literature indicates that heat-killed probiotics reduce inflammation by modulating these receptors and subsequently reducing NF-κB activity [31]. However, MB-2006 appears to have a more direct and potent effect on the inhibition of the NF-κB pathway compared to LAC and LRH. This enhanced efficacy is likely due to the synergistic effects of the bioactive compounds in *Smilax china* L. and the metabolites produced during fermentation. The significant reduction in the p-p65/p65 ratio and lower levels of inflammatory cytokines in MB-2006-treated cells compared to LAC and LRH (Figure 2, Figure 3 and Figure 4) suggest that MB-2006 has a stronger inhibitory effect on NF-κB activation.

During the preparation of protein samples for Western blot analysis, it is crucial to isolate both cytosolic and nuclear fractions to accurately detect transcription factors such as NF-κB, specifically its phosphorylated form (p-p65). NF-κB translocates from the cytosol to the nucleus upon activation, where it regulates the transcription of target genes involved in inflammatory responses. However, in our sample preparation, the nuclear fraction was not included, and the Western blot primarily reflects the cytosolic levels of p-p65. This likely resulted in the detection of faint p-p65 bands on the Western blot, as the nuclear p-p65 was not represented. To ensure comprehensive detection of NF-κB activity, future experiments should include both cytosolic and nuclear fractions in the sample preparation. This approach is expected to provide a complete analysis of NF-κB activation and translocation, thereby improving the accuracy of the results. We acknowledge that our sample preparation protocols need to be refined accordingly to enhance the detection and interpretation of key signaling pathway components [34,40].

In summary, the mechanism by which MB-2006 inhibits the NF-κB pathway involves the suppression of IKK activity, prevention of IκBα degradation, and subsequent inhibition of NF-κB translocation to the nucleus. This mechanism is more effective than that of heat-killed probiotics (LAC and LRH), likely due to the synergistic effects of the bioactive components in MB-2006. This enhanced inhibition leads to a significant reduction in pro-inflammatory cytokine production, thereby alleviating AD symptoms. These findings underscore the potential of MB-2006 as a potent therapeutic alternative for treating atopic dermatitis.

## Data Availability

Data is contained within the article.

## References

[1] Di Marzio L., Centi C., Cinque B., Masci S., Giuliani M., Arcieri A., Zicari L., De Simone C., Cifone M.G. (2003). Effect of the lactic acid bacterium Streptococcus thermophilus on stratum corneum ceramide levels and signs and symptoms of atopic dermatitis patients. Exp. Dermatol..

[2] Kondo H., Ichikawa Y., Imokawa G. (1998). Percutaneous sensitization with allergens through barrier-disrupted skin elicits a Th2-dominant cytokine response. Eur. J. Immunol..

[3] Taïeb A. (1999). Hypothesis: From epidermal barrier dysfunction to atopic disorders. Contact Dermat..

[4] Lee J.Y., Kim Y., Kim J.-I., Lee H.-Y., Moon G.-S., Kang C.-H. (2022). Improvements in Human Keratinocytes and Antimicrobial Effect Mediated by Cell-Free Supernatants Derived from Probiotics. Fermentation.

[5] Tolosa L., Donato M.T., Gómez-Lechón M.J., Vinken M., Rogiers V. (2015). General Cytotoxicity Assessment by Means of the MTT Assay. Protocols in In Vitro Hepatocyte Research.

[6] Guenounou M. (1998). Cytokines and allergic response. Ann. Biol. Clin..

[7] Wu S., Pang Y., He Y., Zhang X., Peng L., Guo J., Zeng J. (2021). A comprehensive review of natural products against atopic dermatitis: Flavonoids, alkaloids, terpenes, glycosides and other compounds. Biomed. Pharmacother..

[8] Man G., Hu L.-Z., Elias P.M., Man M.-Q. (2018). Therapeutic Benefits of Natural Ingredients for Atopic Dermatitis. Chin. J. Integr. Med..

[9] Challinor V.L., Parsons P.G., Chap S., White E.F., Blanchfield J.T., Lehmann R.P., De Voss J.J. (2012). Steroidal saponins from the roots of Smilax sp.: Structure and bioactivity. Steroids.

[10] Huang H.-L., Lu Z.-Q., Chen G.-T., Zhang J.-Q., Wang W., Yang M., Guo D.-A. (2007). Phenylpropanoid-Substituted Catechins and Epicatechins from Smilax china. Helv. Chim. Acta.

[11] Ha Y., Lee W.-H., Kim J.K., Jeon H.-K., Lee J., Kim Y.-J. (2022). Polyopes affinis Suppressed IFN-γ- and TNF-α-Induced Inflammation in Human Keratinocytes via Down-Regulation of the NF-κB and STAT1 Pathways. Molecules.

[12] Kim H.J., Baek J., Lee J.R., Roh J.Y., Jung Y. (2018). Optimization of Cytokine Milieu to Reproduce Atopic Dermatitis-related Gene Expression in HaCaT Keratinocyte Cell Line. Immune Netw..

[13] Hill C., Guarner F., Reid G., Gibson G.R., Merenstein D.J., Pot B., Morelli L., Canani R.B., Flint H.J., Salminen S. (2014). The International Scientific Association for Probiotics and Prebiotics consensus statement on the scope and appropriate use of the term probiotic. Nat. Rev. Gastroenterol. Hepatol..

[14] Lizardo M.V.P., Tavaria F.K., Brandelli A. (2022). Chapter 19—Probiotics and skin health. Probiotics.

[15] Lee J.Y., Park J.Y., Jeong Y., Kang C.H. (2023). Anti-Inflammatory Response in TNFα/IFNγ-Induced HaCaT Keratinocytes and Probiotic Properties of Lacticaseibacillus rhamnosus MG4644, Lacticaseibacillus paracasei MG4693, and Lactococcus lactis MG5474. J. Microbiol. Biotechnol..

[16] Vinderola G., Sanders M.E., Cunningham M., Hill C. (2023). Frequently asked questions about the ISAPP postbiotic definition. Front. Microbiol..

[17] Piqué N., Berlanga M., Miñana-Galbis D. (2019). Health Benefits of Heat-Killed (Tyndallized) Probiotics: An Overview. Int. J. Mol. Sci..

[18] Taverniti V., Guglielmetti S. (2011). The immunomodulatory properties of probiotic microorganisms beyond their viability (ghost probiotics: Proposal of paraprobiotic concept). Genes Nutr..

[19] Seung-Je L., Eun-Gyeong L., Ga-Yeon K., Mi-Ji J., Sang-Yong K., Young-Min K., Geun Y. (2017). Study of Anti-atopic Dermatitis Effects of Juice of Raphanus sativus var in HaCaT Cell Line. KSBB J..

[20] Yan F., Li F., Liu J., Ye S., Zhang Y., Jia J., Li H., Chen D., Mo X. (2020). The formulae and biologically active ingredients of Chinese herbal medicines for the treatment of atopic dermatitis. Biomed. Pharmacother..

[21] Kim K., Kim H., Sung G.Y. (2022). An Interleukin-4 and Interleukin-13 Induced Atopic Dermatitis Human Skin Equivalent Model by a Skin-On-A-Chip. Int. J. Mol. Sci..

[22] Albanesi C., Scarponi C., Sebastiani S., Cavani A., Federici M., Sozzani S., Girolomoni G. (2001). A cytokine-to-chemokine axis between T lymphocytes and keratinocytes can favor Th1 cell accumulation in chronic inflammatory skin diseases. J. Leukoc. Biol..

[23] Ramanathan S., de Kozak Y., Saoudi A., Goureau O., Van der Meide P.H., Druet P., Bellon B. (1996). Recombinant IL-4 aggravates experimental autoimmune uveoretinitis in rats. J. Immunol..

[24] Hwang D.H., Koh P.O., Kang C., Kim E. (2021). Rosa davurica Pall. improves DNCB-induced atopic dermatitis in mice and regulated TNF-Alpa/IFN-gamma-induced skin inflammatory responses in HaCaT cells. Phytomedicine.

[25] Iwaszko M., Biały S., Bogunia-Kubik K.A.-O. (2021). Significance of Interleukin (IL)-4 and IL-13 in Inflammatory Arthritis. Cells.

[26] Datsi A.A.-O., Steinhoff M.A.-O., Ahmad F.A.-O., Alam M.A.-O., Buddenkotte J.A.-O. (2021). Interleukin-31: The "itchy" cytokine in inflammation and therapy. Eur. J. Allergy Clin. Immunol..

[27] Nygaard U., Hvid M., Johansen C., Buchner M., Fölster-Holst R., Deleuran M., Vestergaard C. (2016). TSLP, IL-31, IL-33 and sST2 are new biomarkers in endophenotypic profiling of adult and childhood atopic dermatitis. J. Eur. Acad. Dermatol. Venereol..

[28] Iyer S.S., Cheng G. (2012). Role of interleukin 10 transcriptional regulation in inflammation and autoimmune disease. Crit. Rev. Immunol..

[29] Oh J.H., Kim S.H., Kwon O.K., Kim J.H., Oh S.R., Han S.B., Park J.A.-O., Ahn K.S. (2022). Purpurin suppresses atopic dermatitis via TNF-α/IFN-γ-induced inflammation in HaCaT cells. Int. J. Immunopathol. Pharmacol..

[30] Yang C.-C., Hung Y.-L., Ko W.-C., Tsai Y.-J., Chang J.-F., Liang C.-W., Chang D.-C., Hung C.-F. (2021). Effect of Neferine on DNCB-Induced Atopic Dermatitis in HaCaT Cells and BALB/c Mice. Int. J. Mol. Sci..

[31] Liu T., Zhang L., Joo D., Sun S.-C. (2017). NF-κB signaling in inflammation. Signal Transduct. Target. Ther..

[32] Choi J.H., Lee G.H., Jin S.W., Kim J.Y., Hwang Y.A.-O., Han E.A.-O., Kim Y.A.-O., Jeong H.A.-O. (2021). Impressic Acid Ameliorates Atopic Dermatitis-Like Skin Lesions by Inhibiting ERK1/2-Mediated Phosphorylation of NF-κB and STAT1. Int. J. Mol. Sci..

[33] Karin M., Hunter T. (1995). Transcriptional control by protein phosphorylation: Signal transmission from the cell surface to the nucleus. Curr. Biol..

[34] Karin M., Ben-Neriah Y. (2000). Phosphorylation Meets Ubiquitination: The Control of NF-κB Activity. Annu. Rev. Immunol..

[35] Parada Venegas D., De la Fuente M.K., Landskron G., González M.J., Quera R., Dijkstra G., Harmsen H.J.M., Faber K.N., Hermoso M.A. (2019). Short Chain Fatty Acids (SCFAs)-Mediated Gut Epithelial and Immune Regulation and Its Relevance for Inflammatory Bowel Diseases. Front. Immunol..

[36] Jenab A., Roghanian R., Emtiazi G.A.-O. (2020). Bacterial Natural Compounds with Anti-Inflammatory and Immunomodulatory Properties (Mini Review). Drug Des. Devel. Ther..

[37] Lee J.Y., Kang J.-H., Jung Y.-R., Kang C.-H. (2023). Lactobacillus gasseri MG4247 and Lacticaseibacillus paracasei MG4272 and MG4577 Modulate Allergic Inflammatory Response in RAW 264.7 and RBL-2H3 cells. Probiotics Antimicrob. Proteins.

[38] Rusu E., Enache G., Cursaru R., Alexescu A., Radu R., Onila O., Cavallioti T., Rusu F., Posea M., Jinga M. (2019). Prebiotics and probiotics in atopic dermatitis. Exp. Ther. Med..

[39] Li Y., Won K.J., Kim D.Y., Kim H.B., Kang H.M., Lee S.Y., Lee H.A.-O. (2021). Positive Promoting Effects of Smilax China Flower Absolute on the Wound Healing/Skin Barrier Repair-Related Responses of HaCaT Human Skin Keratinocytes. Chem. Biodivers..

[40] Choi C.Y., Kim Y.H., Oh S., Lee H.J., Kim J.H., Park S.H., Kim H.J., Lee S.J., Chun T.A.-O. (2017). Anti-inflammatory potential of a heat-killed Lactobacillus strain isolated from Kimchi on house dust mite-induced atopic dermatitis in NC/Nga mice. J. Appl. Microbiol..

